# Neoadjuvant immunotherapy in primary and metastatic colorectal cancer

**DOI:** 10.1093/bjs/znab342

**Published:** 2021-10-25

**Authors:** A Kanani, T Veen, K Søreide

**Affiliations:** 1 Department of Gastrointestinal Surgery, Stavanger University Hospital, Stavanger, Norway; 2 Department of Gastrointestinal Surgery, Gastrointestinal Translational Research Unit, Stavanger University Hospital, Stavanger, Norway; 3 Department of Clinical Medicine, University of Bergen, Bergen, Norway

## Abstract

**Background:**

Colorectal cancer (CRC) is the second most common solid organ cancer. Traditional treatment is with surgery and chemotherapy. Immunotherapy has recently emerged as a neoadjuvant therapy that could change treatment strategy in both primary resectable and metastatic CRC.

**Methods:**

A literature review of PubMed with a focus on studies exploring upfront immunotherapy in operable CRC, either for primary resectable stage I–III cancers or for (potentially) operable liver metastasis.

**Results:**

Immune checkpoint blockade by the programmed cell death 1 (PD-1) receptor inhibitors nivolumab and pembrolizumab and the cytotoxic T cell-associated protein 4 (CTLA-4) inhibitor ipilimumab has shown good results in both early-stage and advanced CRC. The effects of immune checkpoint inhibitors have so far been demonstrated in small phase I/II studies and predominantly in treatment-refractory stage IV disease with defect Mismatch repair (dMMR). However, recent data from phase I/II (NICHE-1) studies suggest an upfront role for immunotherapy in operable stage I–III disease. By blocking crucial immune checkpoints, cytotoxic T cells are activated and release cytotoxic signals that initiate cancer cell destruction. The very high complete response rate in dMMR operable CRC with neoadjuvant immunotherapy with nivolumab and ipilimumab, and even partial pathological response in some patients with proficient MMR (pMMR) CRC, calls for further attention to patient selection for neoadjuvant treatment, beyond MMR status alone.

**Conclusion:**

Early data on the effect of immunotherapy in CRC provide new strategic thinking of treatment options in CRC for both early-stage and advanced disease, with prospects for new trials.

## Teaser

Immunotherapy has been demonstrated to be effective in subgroups of patients with colorectal cancer. Molecular subtypes may provide an opportunity for new strategic treatment plans, in addition to surgery and chemotherapy.

## Introduction

Colorectal cancer (CRC) is the second most common solid organ cancer in both sexes, representing a considerable global health burden. Prognosis has improved across all cancer stages over the last decade through improvements in surgical and medical oncological management. Targeted therapies with anti-epidermal growth factor receptor (EGFR) drugs (for example, cetuximab in *RAS* non-mutants) or anti-vascular endothelial growth factor (VEGF) treatment (for example, bevacizumab for *RAS* mutants) have become the standard of care^1^. However, a considerable number of patients treated with curative intent will develop metastases and eventually die of disseminated treatment-resistant disease. Effective chemotherapy regimens, combined with biological agents, have increased the median survival to > 3 years for patients with stage IV disease. Increased resection rates for hepatic metastasis are associated with 5-year overall survival approaching 50 per cent after liver surgery^2^, although only about 1 in 5 patients can be offered metastatic surgery. Despite the progress made, < 15 per cent of all patients with stage IV CRC are alive at 5 years from diagnosis^3^.

In parallel with improved management, the molecular pathways in CRC have been described in more detail, with proposed implication for personalized therapy^4^. Avoiding immune destruction is a cancer hallmark^5^ and is facilitated through several mechanisms, including *KRAS* mutations in CRC^6^. However, an immunogenic subtype of CRC has been found to be associated with a favourable outcome and a remarkable and durable response to immunotherapy^7^.

**Fig. 1:**
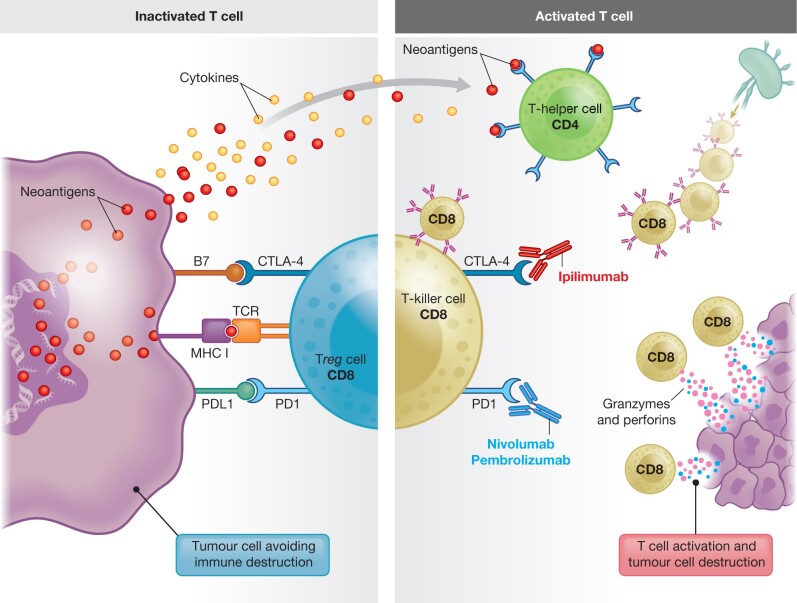
Immunotherapy by immune checkpoint blockage in colorectal cancer Simplistic overview of mechanisms of T cell activation/inactivation in the tumour microenvironment. The inactive state of T cells in immune ‘cold’ tumours (on the left). Major histocompatibility complex (MHC)-T cell receptor (TCR)-dependent signalling demonstrates immune evasion by tumour cells expressing the inhibitory ligand PD-L1, which binds to PD-1 on T cells (and B7 molecules) which bind to CTLA-4. Cancer cell engagement with inhibitory ligands (against PD-1 and CTLA-4) prevents cytotoxic killing of tumour cells. PD-L1 binding initiates a signalling cascade that stimulates conversion of effector T cells to regulatory T cells (Tregs). Tumours in the active state (immune ‘hot’ tumours on right side of panel). CD8^+^ T cells recognize tumour-associated antigens expressed on MHC class I on tumour cells via the TCR, which results in cytotoxic killing of tumour cells via release of granzymes and perforins. Inhibitory ligands against PD-1 and CTLA-4 are blocked by immune checkpoint inhibitors, allowing active T cell function towards cancer cells. Several associated mechanisms are involved, in addition, including dendritic cells that serve as a biologic immune intermediate for neoantigen delivery, with an ability to augment the immune response through cytokine release. CTLA-4, cytotoxic T lymphocyte-associated protein 4; PD-1, programmed cell death 1; PD-L1, PD-1 ligand.

The immunogenic subtype of CRC is related to a defective mismatch repair (dMMR) system and associated with high-frequency microsatellite instability (MSI-H). The dMMR/MSI-H cancers have long been associated with strong lymphocytic infiltration in and around the tumour^8^^,^^9^. The dMMR tumours have a high tumour mutational burden and an abundance of neoantigens^7^^,^^10^, the latter contributing to an activated immune cell response and antitumour activity^11–13^. However, not all immune cells in the tumour microenvironment are activated against the tumour cell^14^, and immune escape is an essential part of clonal cancer evolution^15^^,^^16^. Metaphorically speaking, the cancer–immune cell interaction renders tumours either ‘immune cold’ or ‘immune altered’, or ‘immune hot’^17^, reflecting an opportunity for T cell activation by immune checkpoint blockade to promote cancer cell destruction (*Fig. 1*). While dichotomization of the immune response is unlikely to be biologically correct, there is emerging interest in mechanisms that can activate immune-dormant tumours. The dormant nature is usually attributed to exhausted T cells which may be activated by immune checkpoint inhibitors. While immune checkpoint inhibition is most effective in dMMR cancers^18^, the involved mechanisms are currently incompletely understood and may not be related to MMR status alone^7^. Indeed, some microsatellite-stable (MSS) cancers are hypermutated, based on specific genetic mutations such as polymerase epsilon (*POLE*) and polymerase delta (*POLD1*)^19^^,^^20^. POLE and POLD1 are proofreading domains of the DNA which repair genetic defects. Mutations in these genes are found in < 4 per cent of CRCs^20^. However, when present, *POLE*/*POLD1-mutated cancers* are associated with a high tumour mutational burden; hence, their use has also been suggested in predicting efficacy of immunotherapy^19–22^. Of note, recent studies have suggested that the rate of tumour mutational burden in MSS cancers has relevance to immunotherapy response, with a higher tumour mutational burden showing response to immunotherapy^7^^,^^23–26^. Several relevant genes have been associated with a high tumour mutational burden (including *ARID1A*, *RNF43*, *BRAF and KM2B* in microsatellite instability (MSI) cancers) and may help select MSS tumours for immunotherapy^25–27^. Some studies have also suggested using tumour mutational burden as a biomarker for measuring response to immunotherapy. Measuring tumour mutational burden in MSS cancers may be helpful in selecting patients who would benefit from immunotherapy. However, it is currently not clear which test works best for selecting patients suitable for immunotherapy. In a systematic review, concurrent occurrence of MSI, high tumour mutational burden and positive programmed cell death ligand 1 (PD-L1) expression was found in only 12.8 per cent of CRCs^23^. MSI and tumour mutational burden had the greatest overlap, with PD-L1 positive expression occurring in > 40 per cent of cases with no associated MSI or tumour mutational burden. Hence, relying on any one single test may not capture all patients who may eventually have a response and benefit from immunotherapy. Notably, most studies on treatment-refractory metastatic CRC have relied on dMMR subtypes for inclusion in trials^28–30^.

There is considerable interest in using immunotherapy beyond the metastatic setting, supported by the remarkable progress made in several other solid organ cancers with use of immune checkpoint inhibitors^31^. This review describes the potential influence of immunotherapy on operable and advanced CRC and possible related changes to clinical practice and research.

## Mechanisms of action of immunotherapy in CRC with dMMR

The Food and Drug Administration (FDA) first approved the use of immunotherapy drugs for metastatic CRC in 2017^18^^,^^32–34^. Recent trials have suggested a potential change in management of both operable and advanced CRC with use of immune checkpoint inhibitors^35^^,^^36^. The currently approved drugs nivolumab and pembrolizumab block the programmed cell death 1 (PD-1) receptor, whereas ipilimumab blocks cytotoxic T cell-associated protein 4 (CTLA-4) (*Fig. 1*). Several other tumour–immune cell interactions are important for immune system activation or depression in cancer, including major histocompatibility complex (MHC) class I interaction and signalling with the T cell receptor (TCR). Several of these tumour–immune cell interactions are under investigation for potential therapeutic use^7^^,^^31^^,^^37^. Recent data suggest that downregulation of MHC class II protein is associated with a poorer immune response and scant number of T cells surrounding the tumour, with an opportunity to activate an immune response through MHC class II^38^^,^^39^. Notably, the mechanisms underlying the tumour–immune cell environment are complex and include several possible co-evolving pathways along the adenoma–carcinoma–metastasis pathway in CRC^11^^,^^15^^,^^40–42^.

PD-1 and/or CTLA-4 checkpoint inhibition has proven to be highly effective for the treatment of patients with advanced dMMR CRC^18^^,^^32^. Patients with dMMR/MSI-H cancers have a high tumour mutational burden due to a very high number of missense mutations, including indels and frameshift mutations in the tumour genome. As a result, these genetic alterations produce a high number of neoantigens (or alternatively referred to as neopeptides or neoepitopes) that are tumour-specific and may be recognized by immune cells as foreign, hence eliciting an immune response with an abundance of T cells in the tumour surroundings. The abundance and type of immune cells are related to clinical outcome^8^, with better disease-specific survival rates in patients with a high number of tumour-infiltrating lymphocytes (TILs)^9^^,^^43^. However, in some cancers, T cells may be inactive due to binding of the PD-1 receptor to the tumour cell ligand PD-L1. In this instance, cytotoxic T cells are converted to regulatory T cells (Tregs) which do not exert a destructive effect on tumour cells. Through blockade of PD-1 (for example, pembrolizumab), cytotoxic T cells are activated and release cytotoxic signals that initiate cancer cell destruction (*Fig. 1*).

## Immunotherapy according to consensus molecular subtypes

**Fig. 2:**
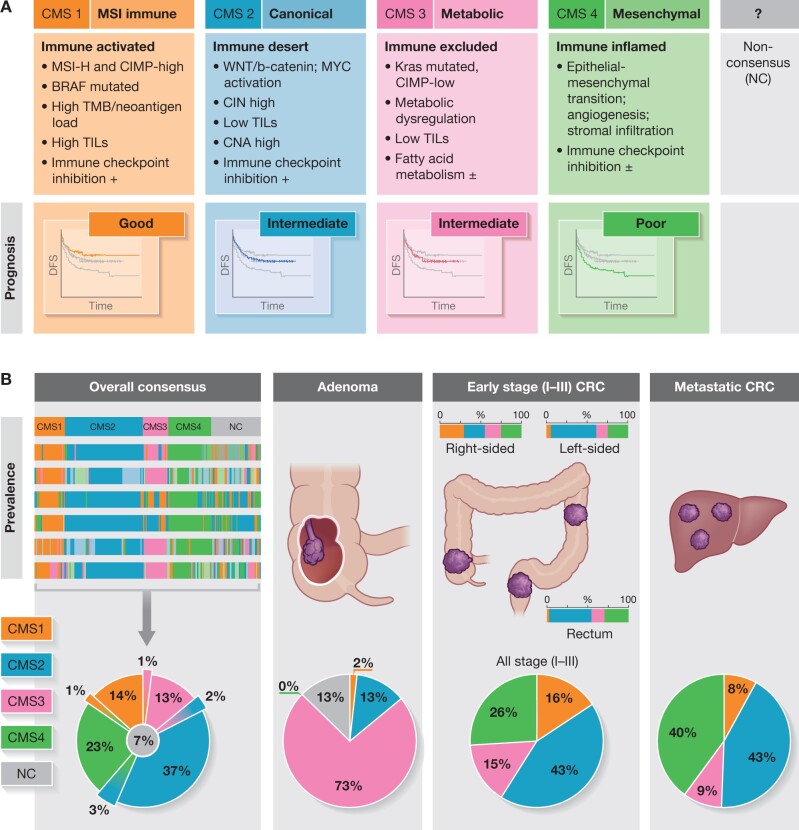
Context- and stage-based prevalence of consensus molecular subtypes in early-stage and advanced colorectal cancer Depicted are the main features of each of the four subtypes (**a**) and the context-dependent distribution across disease stages (**b**). The actual CMS distribution may vary between trials and studies, based on inclusion criteria, with direct impact on study population outcomes. Data are approximated from several different studies.

Classification based on consensus molecular subtypes (CMS)^44^ broadly defines disease into four groups (*Fig. 2*) with clinical relevance^4^^,^^45–47^. CMS type 1 (or ‘MSI immune’) is particularly suited for immunotherapy, based on the descriptive characteristics of such cancers^18^^,^^48^^,^^49^. Hence, testing for specific molecular traits and measuring immune cell infiltration have become important for both prognostic and predictive purposes in CRC^9^^,^^50–52^. For patients with localized CRC (stages I–III) with dMMR (usually around 15–20 per cent of all CRCs), overall prognosis is better than that for patients with proficient mismatch repair (pMMR) tumours^9^^,^^53^. However, dMMR CRC with metastasis has a very poor prognosis. Metastatic tumours with MSI/dMMR are most often driven by associated *BRAF* mutation^54^, and such metastatic dMMR tumours have poor response to chemotherapy and an overall worse prognosis^55^. Hence, molecular features such as MSI and *KRAS and BRAF* mutations have clear clinical implications and have become essential predictors beyond regular image-based tumour staging.

**Fig. 3:**
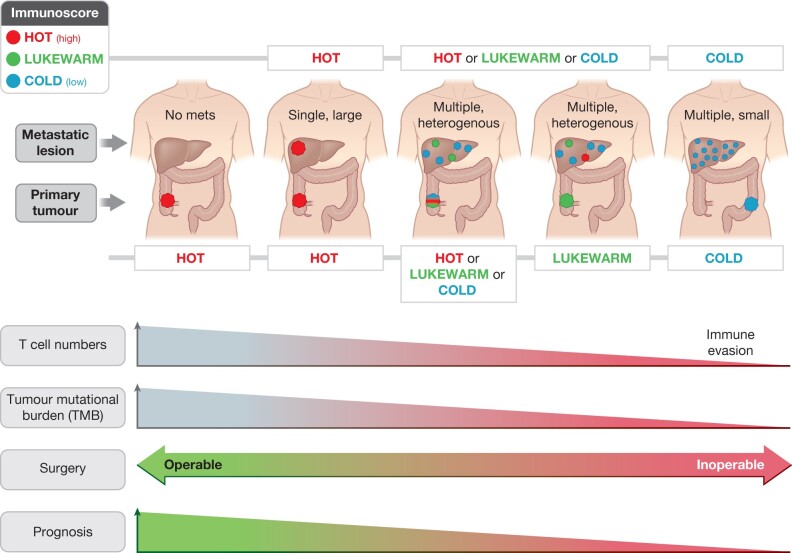
Variation in the immune landscape from early- to advanced-stage CRC The model of immune ‘hot’ and ‘cold’ tumours across the spectrum of primary and stage IV disease is illustrated, based on^63^. Several other factors are involved, including age and sex of patient, tumour sidedness (with more dMMR in right-sided colon cancers being immune hot). Patterns of clinical scenarios associated with the immune profile are emerging such as association of multiple small disseminated liver metastases found in immune ‘cold’ tumours. The clinical presentation of CRC, together with mismatch repair status, immune cell quantification, and molecular features such as tumour mutational burden, may help delineate appropriate and personalized use of immunotherapy and the design of new trials.

However, as demonstrated in several subsequent studies, the CMS groups are not confined within stages and are context-driven in terms of CMS distribution across stage categories of CRC (*Fig. 2b*). Of note, the initial consensus series failed to categorize 7 per cent (with a further 6 per cent having mixed categories) of patients determined as ‘non-categorical’ (NC; *Fig. 2*)^44^. Furthermore, studies have shown that CMS distribution is variable across stages of carcinogenesis (*Fig. 2b*), with a low prevalence of CMS1 in adenomas^56^. A variable prevalence of CMS1 is found across clinical series in stages I–III, with about 16 per cent across the entire colorectum^57^, but with > 25 per cent prevalence for colon cancers and a corresponding lower rate in the rectum. In metastatic (stage IV) disease, a variable CMS1 rate of around 8 per cent has been reported^58^. The distribution of CMS categories largely varies between right- or left-sided colonic cancers and rectal cancers (*Fig. 2b*). Hence, the distribution of the ‘MSI immune’ type is more frequently found in right-sided colonic cancers (up to 25 per cent), but relatively rarely in metastatic CRC (about 8 per cent). Immunotherapy benefit is not solely related to CMS1. As demonstrated in *Fig. 2*, CMS2 and CMS3 generally show an intermediate response to immunotherapy, whereas CMS4 has a poor response. *KRAS* mutations are reported in < 15 per cent of liver metastases overall, yet about 25–40 per cent of patients with resected liver metastases harbour *KRAS* mutation^59–61^. Furthermore, MSI and mismatch repair deficiencies are found more frequently in young-onset CRC and in the elderly with CRC. It seems that rather than categorically placing patients into individual subtypes, the spectrum of immune cell responses demonstrates variation from primary to metastatic tumour^62^, with some being immune ‘hot’ and others ‘altered’ or ‘cold’ (*Fig. 3*)^63^. Therefore, the number of factors involved in immune-activated compared to those in immune-deserted cases show overlap and do not have a perfect correlation with mismatch repair testing or the presence of MSI or tumour mutational burden, nor with quantitative measurement of immune cells beyond group levels. Thus, this may be a slight impediment to perfect prognostic and predictive testing for personalized therapy in CRC. Of note, both dMMR and pMMR may have unexpected responses and failures^64^^,^^65^, which are not currently understood.

## Immunotherapy as first-line treatment in metastatic CRC

The efficacy of immunotherapy as second-line treatment in chemotherapy-refractory metastatic CRC was assessed in CheckMate 142^32^^,^^33^. The trial examined the combined effect of nivolumab and ipilimumab for dMMR metastatic CRC and showed high response rates, with progression-free and overall survival at 12 months at 71 per cent and 85 per cent, respectively. This combination treatment also proved to be safe in terms of efficacy. CheckMate 142^32^^,^^33^ therefore demonstrated that immunotherapy had a place in treating patients with mCRC and MSI-H/dMMR where other treatment no longer had an effect.

The randomized, open-label, multicentre KEYNOTE-177 trial^64^ has changed the standard of care for metastatic CRC with dMMR^36^. The study included 307 patients with metastatic CRC and MSI-H/dMMR. In this single-agent immunotherapy trial, progression-free survival was almost twice as long as that in the pembrolizumab group, compared to the chemotherapy group (median progression-free survival of 16.5 months for pembrolizumab *versus* 8.2 months for chemotherapy). Duration of response was also longer in the pembrolizumab group, with 84 per cent of patients still having partial or complete response 24 months after therapy initiation, *versus* 33 per cent of patients in the chemotherapy group. The complete response rate was also higher in the pembrolizumab group, compared to the chemotherapy group^66^. With fewer severe complications in the pembrolizumab group, safety of the drug was also demonstrated, but more immune-mediated adverse events were recorded, as expected. Therefore, this study shows pembrolizumab to be superior to chemotherapy for patients with MSI-H/dMMR metastatic CRC , with longer progression-free survival and fewer severe adverse events^64^.

## Immunotherapy as upfront treatment in operable CRC

For operable colon cancer (stages I–III), traditional management has been surgery followed by adjuvant chemotherapy for those at high risk of relapse, typically high-risk stage II and all stage III colon cancer. In rectal cancer, neoadjuvant chemotherapy and radiotherapy are more frequently used to improve local control ahead of surgery. However, there has been recent interest in using neoadjuvant chemotherapy in colon cancer patients, particularly in those with locally advanced tumours^67^.

The large multicentre trial (FOxTROT^68^) investigating the role of neoadjuvant chemotherapy in high-risk colon cancer has not yet produced results on final outcomes, including survival data. Currently, the standard of care for operable stage I–III colon cancer is surgery upfront, with adjuvant therapy for patients with high-risk features. Little is known about immunotherapy in operable CRC, although a higher rate (around 25 per cent for colon cancer) of dMMR/MSI-H is expected in this setting, compared to metastatic CRC (dMMR reported to be 6–8 per cent) (*Fig. 2*). Hence, the prospect of a clinical benefit in operable colon cancer should be higher, particularly if guided by the relatively higher frequency of dMMR tumours. Also, rectal cancers with dMMR have a poor response to conventional radiochemotherapy^52^, with anecdotal reports of a very good response to immunotherapy^69^^,^^70^. One study in patients with rectal cancer investigated pretreatment biopsies and found an association between high tumour mutational burden and high T cell infiltration to have a better subsequent response to radiochemotherapy, possibly suggesting that adding immunotherapy may further enhance this effect^71^.

In the NICHE phase I/II trial^65^, the effect of neoadjuvant immunotherapy by doublet immune check blockade was investigated in a cohort of 40 patients with operable colon cancer. Both MSI-H/dMMR (21 patients) and MSS/pMMR (20 patients) cancers were included, of which 35 were evaluable for efficacy and translational endpoints (20 dMMR and 15 pMMR). Patients were given double immune checkpoint blockade with a single dose of ipilimumab and two doses of nivolumab 6 weeks prior to surgery. The treatment was well tolerated and all patients underwent radical resections without delay (meeting the primary endpoint of the trial). Pathological response was observed in 20/20 of dMMR tumours, with 19 major pathological responses (defined as ≤ 10 per cent residual viable tumour on histopathology) and 12 (60 per cent) pathological complete responses. Notable in the NICHE trial, among the pMMR tumours, 4 (3 major and 1 partial responses) of 15 had pathological responses^65^. The difference in response between dMMR and pMMR is mainly attributed to a difference in tumour burden/neoantigens and T cell infiltration. Notably, CD8^+^PD-1^+^ T cell infiltration was predictive of response in pMMR tumours, suggesting that some pMMR tumours are immune-responsive despite not demonstrating dMMR at the molecular level. This demonstrates the complexity of defining ‘hot’ and ‘cold’ tumours with response to therapy beyond simple MMR testing (*Fig. 3*)^63^.

The NICHE-1 study data indicate that neoadjuvant immunotherapy has the potential to become the standard of care for a defined group of colon cancer patients when validated in larger studies. The phase I/II results are corroborated by early reports in rectal cancer with proven MSI-H^69^^,^^70^. The NICHE study also points to an important issue in patient selection beyond dMMR status. One could perceive that absolute T cell counts, or selection by immunoscore, tumour mutational burden analyses, or PD-L1 assessment, may be warranted to identify those patients who would likely benefit from immunotherapy in pMMR cancers. However, correlation among such tests, actual treatment response and effect on overall survival is not yet established. Lastly, one should also question reflexive treatment for all operable dMMR CRC, as a vast majority of these patients will never have recurrence and hence would not benefit from treatment, even if a complete response is obtained. In the setting where resection is planned, the prospects of downstaging in large or bulky tumours may be the most likely benefit of neoadjuvant immunotherapy. However, one could potentially take advantage of the complete response observed in selected patients having non-operative, surveillance-based management, to allow an organ-sparing approach. This would be analogous to the current watch-and-wait trials after complete pathological response to neoadjuvant chemoradiotherapy in rectal cancer^72^^,^^73^.

## Resistance and sustainable effects

Despite the demonstrated effects of immunotherapy, there are concerns regarding immune checkpoint blockade. One concern relates to durability of response and the development of immunotherapy resistance^74^. A hallmark of immunotherapy is presentation of an antigen that the host’s T cell can identify as an intruder cell, and thus initiation of destruction. Hence, immunotherapy resistance can develop from alteration in tumour cells’ lack or ineffective presentation of antigens. Other mechanisms of resistance include aberrant cellular signal transduction and changes in cellular components in the tumour microenvironment, both influencing T cell response to anti-PD-1 therapy. With CTLA-4 blockade resistance, different mechanisms are described. CTLA-4 immune checkpoint is dependent on the costimulatory molecule B7 to induce a response and T cells mediate their effect on CTLA-4 blockade through interferon gamma. Tumour cells develop resistance through changing the expression of the costimulatory molecule B7 oraltered genes which respond to interferon gamma signalling initiated by T cells^7^^,^^74^.

Recently, reports that fecal microbiota transplantation can lead to immune checkpoint inhibitor (ICI) therapy responses in patients previously refractory to therapy suggest that targeting the microbiome may be a viable strategy to reprogramme the tumour microenvironment and augment immunotherapy^75^. A small study^76^ in treatment-refractory melanoma demonstrated the effect of fecal microbiota transplantation in changing the gut flora, and hence reprogramming the tumour microenvironment, to make the cancer more immunogenic. Whether this principle is transferable to other cancers, such as CRC, is currently under investigation.

## Adverse events to immune checkpoint inhibitors

Adverse events from immunotherapy are important as they can increase morbidity, delay surgery in the neoadjuvant setting, and, in worst case scenarios, increase mortality. One review described the colon, liver, lungs, pituitary, thyroid, skin, and, more rarely, the heart and nervous system as affected organs. With ipilimumab (anti-CTLA-4) monotherapy, the most common adverse effect is colitis, whereas nivolumab and pembrolizumab (anti-PD-1) have been found to cause hypothyroidism, rash, and diarrhoea (more commonly than colitis). Combination treatment with both anti-CTLA-4 and anti-PD-1 carry the highest risk of adverse effects, particularly colitis, and often affected multiple organs.

Early concern about immunotherapy-induced colitis preventing subsequent surgery seems unwarranted, with very low numbers (< 1–5 per cent) found across 145 trials^77^, of which only three were conducted in patients with CRC. A slightly higher risk may be found in inflammatory bowel disease-associated cancers, but none have been reported to have treatment-limiting effects^78^. Fatal toxic event rates are very low for ICI (between 0.3 and 1.3 per cent), but with the vast majority of data coming from studies in patients with end-stage melanoma or lung cancer^79^.

## Future direction

Immunotherapy has proven to be highly effective in CRC, with excellent results as first-line treatment in the metastatic setting for dMMR CRC using monotherapy pembrolizumab. Further, the high response rate in operable dMMR colon cancers with preoperative use of double nivolumab and ipilimumab therapy warrants further investigation for its impact on long-term overall survival.

New opportunities for immunotherapy are emerging (*Fig. 3*) that are likely to exploit the immune system to enhance multimodal therapy, improve resection rates, enhance disease control, and eventually improve overall survival for patients. With this, several questions arise with new avenues for research and trials. The current understanding of what makes tumours ‘hot’ and ‘cold’ is incomplete, although data are emerging^17^. The criteria for potential treatment beyond MMR/MSI testing and POLE/POLD1 are unclear, but data from the NICHE trial suggest that immunotherapy may have the potential for complete response in dMMR tumours and at least partial response in some pMMR tumours with higher levels of CD8^+^ T cells^65^.

Currently, the optimal timing and strategy for immunotherapy use are uncertain. Should the immune system be modulated as early as possible to potentially achieve the best oncological results? It is feasible that neoadjuvant immune checkpoint blockade could become the preferred option in operable CRC, if this translates into reduced recurrence and improved overall survival. It is possible that immunotherapy may become an organ-sparing treatment option, if complete response is durable and sustained and translates into equivalent oncological effects to surgery. However, there are currently no data to suggest which patients in stage I–III CRC should benefit from this. Hence, novel criteria for appropriate selection for use of immune checkpoint inhibitors must be developed to show the effects on survival outcomes in larger trials. For metastatic disease, it is obviously of interest to explore the ability of immune checkpoint inhibitors to facilitate conversion from unresectable to resectable disease and to improve survival^2^. Further, in what way immunotherapy belongs to just one or more of the CMS subtypes in CRC is not clear. Further research should therefore explore the clinical impact of immunotherapy in both early-stage and advanced CRC.
